# Prevalence and antimicrobial susceptibility pattern of anorectal and vaginal group B *Streptococci* isolates among pregnant women in Jimma, Ethiopia

**DOI:** 10.1186/s13104-016-2158-4

**Published:** 2016-07-19

**Authors:** Abeba Mengist, Hemalatha Kannan, Alemseged Abdissa

**Affiliations:** Department of Medical Laboratory Science, College of Health Science and Medical Sciences, Debre Markos University, P.O. Box 269, Debre Markos, Ethiopia; Department of Medical Laboratory Science and Pathology, College of Public Health and Medical Sciences, Jimma University, Jimma, Ethiopia

**Keywords:** Group B *Streptococcus* (GBS), Pregnant women, Antimicrobial susceptibility, Ethiopia

## Abstract

**Background:**

*Streptococcus agalactiae* (group B *Streptococcus*, GBS) is the most frequent pathogen isolated from neonates with invasive bacterial disease and responsible for serious infections in newborns such as pneumonia, septicemia and meningitis. Infection is primarily acquired vertically from mothers colonized with GBS. However, the prevalence and antimicrobial susceptibility pattern of GBS among pregnant women in Ethiopia are less studied.

**Methods:**

This cross-sectional study involved 126 pregnant women at 35–37 weeks of gestation attending the antenatal clinic at Jimma University Hospital. Anorectal and vaginal swabs were cultured on to Todd-Hewitt broth medium supplemented with Gentamicin and Nalidixic acid and subsequently sub-cultured on 5 % sheep blood agar followed by identification of isolates based on colonial morphology, Gram stain, catalase reaction, hippurate hydrolysis and Christie, Atkins, Munch-Petersen (CAMP) test, and testing for their susceptibility to antimicrobial agents using the Kirby–Bauer method.

**Results:**

The overall carriage rate of GBS was 19.0 % (24/126), and the rectal and vaginal carrier rates were 14.3 % (18/126) and 10.4 % (13/126), respectively. Concomitant vaginal and anorectal colonization was recorded in 29.2 % (7/24) of the women who were culture positive. All GBS isolates were susceptible to penicillin G, ampicillin, and vancomycin, but a considerable proportion was resistant to clindamycin (3.2 %), erythromycin (6.5 %), ciprofloxacin (9.7 %), ceftriaxone (9.7 %), norfloxacin (12.9 %), cotrimoxazole (29 %), and tetracycline (45.2 %).

**Conclusion:**

This study reveals high carriage rate of GBS among pregnant women compared to some previous studies in Ethiopia. However, further epidemiological investigations should be done in different parts of the country in order to know the actual GBS colonization rate of pregnant women and to consider the possibility of implementing prophylactic treatment to prevent potential adverse maternal and neonatal outcomes. Future studies should be conducted to reveal serotype distributions of GBS in this community.

## Background

*Streptococcus agalactiae* (group B *Streptococcus*, GBS) is the most frequent pathogen isolated from neonates with invasive bacterial disease and responsible for serious infections in newborns such as pneumonia, septicemia and meningitis [1–3]. GBS is also associated with significant maternal peripartal disease, including bacteremia, endocarditis, chorioamnionitis, endometritis, urinary tract infections, arthritis, and responsible for serious bacterial illness and deaths in nonpregnant women with underlying diseases and in elderly adults [4–7]. GBS can also pass through the cervix without causing serious cervicitis, and cross-intact amniotic membrane causing amnionitis thereby infecting the foetus within the uterus [4].

The lower gastrointestinal tract is considered as the most likely human reservoir of GBS) with a secondary spread to the genitourinary tract. Pregnant women who are GBS carriers have the potential to transmit the organism to their newborns [8]. Because vaginal colonization is believed to be responsible for the intrapartum exposure of neonates to GBS, it is important clinically to identify these women before delivery. In the absence of maternal chemoprophylaxis, up to 50 % of neonates born to colonized women acquire GBS colonization and 1–2 % of these neonates acquire invasive disease [9]. The rate of GBS colonization during pregnancy varies among different countries [10]. A review that has specifically looked at the prevalence of maternal colonization with GBS indicated the estimated mean prevalence of GBS colonization was 17.9 % overall and was the highest in Africa (22.4 %) followed by the Americas (19.7 %) and Europe (19.0 %). However, Studies from Southeast Asia had the lowest estimated mean prevalence (11.1 %) [10]. In this review, significant heterogeneity was noted across and within regions. Differences in the timing of specimen collection in pregnancy, selective culture methods, and study sample size did not explain the heterogeneity [10]. In Ethiopia, the study by Muhammed et al. showed that GBS colonization rates (20.6 %) were higher than reported by Lakew et al. (7.2 %) [11, 12].

Prevention guidelines for perinatal GBS disease were issued by Centers for Disease Control and Prevention (CDC) in 1996 [13]. The recommendations were revised in 2002, when guidelines for prevention of vertical transmission, through routine screening (culture of vaginal and anorectal secretions between the 35th and 37th gestational week) and intrapartum antibiotic prophylaxis of the colonized women was definitely established. Positivity for GBS colonization found during this period of gestation is highly predictive of the presence of GBS at a time of childbirth [14].

In Ethiopia, few studies have been conducted on GBS [11, 12, 15–17] although infant mortality is high in this population. Until today, there are no public health policies or strategies in Ethiopia aimed at the reduction of GBS neonatal infection. This study, therefore, aimed at determining the proportion of pregnant women colonized with GBS and antimicrobial susceptibility pattern of the isolates at Jimma University Hospital.

## Methods

### Study area and participants

The present cross-sectional study included 126 pregnant women (from 35 to 37 weeks of gestation) who were attending the antenatal clinic at Jimma University Hospital, Jimma, Ethiopia for routine antenatal visits, during the period from July 2012 and December 2012. Jimma is located 355 km southwest of Addis Ababa. At this institution, it is not a common practice to determine colonization status, and hence, not common practice either, to offer prophylaxis.

### Data collection

Data was collected by the attending midwifery nurse using a well-structured questionnaire designed to obtain socio-demographic data and other relevant information such as age, occupation, educational level and gravidity from participants following their written informed consent and the ethical approval of the study from Jimma University ethics reviewed board. All consenting mothers with gestation age 35–37 weeks were included. Participants with a history of using antibiotic(s) 2 weeks prior to the study were excluded from the study.

### Sample collection and laboratory procedure

Samples were collected by the attending midwifery nurse from the lower third of the vagina and rectum of all participants at 35–37 weeks of gestation using a sterile cotton swab and transported in Amies transport media with charcoal (Oxoid, UK) to the microbiology laboratory. Thereafter, specimens were inoculated in Todd-Hewitt broth (Oxoid, UK), an enrichment medium for GBS, supplemented with 8 µg/ml gentamicin and 15 µg/ml nalidixic acid (Biomerieux, France) to prevent the growth of contaminants. The broth cultures were incubated at 37 °C aerobically. After 18–24 h of incubation, specimens were sub cultured onto 5 % sheep blood agar plates (Oxoid, UK) and re-incubated at the recommended incubation conditions. Observation of cultures was conducted at 24 h and all negative culture plates were re-incubated for an additional 18–24 h and then re-examined. Suspicious GBS colonies were identified by colony morphology, gram stain and biochemical tests such as catalase, sodium hippurate hydrolysis and Christie, Atkins, Munch-Petersen (CAMP) factor positivity’s. Finally, antimicrobial susceptibility of all GBS isolates was determined by Kirby–Bauer disk diffusion method on Mueller–Hinton agar supplemented with 5 % sheep blood according to the guideline recommendations of the Clinical and Laboratory Standards Institute [18]. The following antimicrobial discs and concentrations (in brackets) were used: penicillin G (10 IU), ampicillin (10 μg), clindamycin (2 μg), erythromycin (15 μg), vancomycin (30 μg), ciprofloxacin (5 μg), ceftriaxone (30 μg), cotrimoxazole (25 μg), tetracycline (30 µg), and norfloxacin (10 µg). The plates were incubated at 37 °C for 20–24 h. The results were interpreted according to Clinical and Laboratory Standards Institute guidelines [18].

### Statistical analysis

All statistical calculations were done using SPSS for windows version 16. Descriptive statistics were computed to determine the rate of GBS and other variables. The relationships between GBS colonization and various risk factors were tested using the Chi square test. A p value of ≤0.05 was considered indicative of a statistically significant.

## Results

### Socio-demographic and isolation rate

A total of 126 pregnant women were included within this study. The age of the women ranged from 16 to 40 years with mean age of 26.5 years (SD ±4.9). The majority (37.3 %) of the participants were between the ages of 25–29 years. Among the 126 pregnant women screened, 24 (19 %) were colonized with GBS (Table 1). The rectal and vaginal carrier rates were 18 (14.3 %) and 13 (10.3 %), respectively. Of the 24 carriers, GBS was isolated only from the rectum in 11 (45.8 %), from the vagina in 6 (25 %), and concomitant vaginal and anorectal colonization was recorded in 7 (29.2 %) of the women.

**Table 1:** Isolation sites and number of GBS carriers (n = 24)

Isolation site	N (%)
Only rectum	11 (8.7)
Only vagina	6 (4.8)
Both vagina and rectum	7 (5.6)
Total	24 (19)

Sociodemographic and obstetric characteristics of the pregnant women are described in Table 2. No associations were detected between risk factors and GBS colonization of the study subjects but the study was not powered to assess these.

**Table 2:** Association between socio-demographic, obstetric factors and GBS colonization among pregnant women (n = 126)

Variables	Total, n (%)	GBS colonization		P value*
		Colonized, n (%)	Non-colonized, n (%)	
Age in years				
15–19	7 (5.6)	3 (12.5)	4 (3.9)	0.43
20–24	36 (28.6)	9 (37.5)	27 (26.5)	
25–29	47 (37.3)	7 (29.2)	40 (39.2)	
30–34	25 (19.8)	4 (16.7)	21 (20.6)	
35–39	9 (7.1)	1 (4.2)	8 (7.8)	
40–44	2 (1.6)	0 (0)	2 (2.0)	
Educational level				
Illiterate	17 (13.5)	6 (25)	11 (10.8)	0.32
Primary	27 (21.4)	5 (20.8)	22 (21.6)	
Secondary	58 (46)	9 (37.5)	49 (48.0)	
Post-secondary	24 (19)	4 (16.7)	20 (19.6)	
Occupation				
House wife	81 (64.3)	20 (83.3)	61 (59.8)	0.18
Employed	34 (27)	3 (12.5)	31 (30.4)	
Merchant	3 (2.4)	0 (0)	3 (2.9)	
Student	8 (6.3)	1 (4.2)	7 (6.9)	
Gravidity				
Primigravida	38 (30.2)	5 (20.8)	33 (32.4)	0.27
Multigravida	88 (69.8)	19 (79.2)	69 (67.6)	
Total	126 (100)	24 (19)	102 (81)	

* p value: chi squared test comparing colonized and non-colonized pregnant women

### Antimicrobial susceptibility pattern of GBS isolates

The susceptibility patterns of GBS (n = 31) isolated from pregnant women against ten antimicrobial agents are presented in Table 3. All GBS isolates were uniformly susceptible to penicillin G, ampicillin, and vancomycin. Relatively, GBS showed low resistance to clindamycin (3.2 %) and erythromycin (6.5 %), and high resistance to cotrimoxazole and tetracycline with 29 and 45.2 %, respectively. Excluding penicillin G, ampicillin, and vancomycin, antibiogram of GBS isolates revealed that only 9 (29 %) isolates were susceptible to all antibiotics tested and 22 (71 %) were resistant to one or more antibiotics tested (data not presented).

**Table 3:** Antimicrobial susceptibility pattern of GBS (n = 31) isolated from pregnant women

Antibiotics	Susceptible (%)	Intermediate (%)	Resistant (%)
Penicillin G	100	–	0
Ampicillin	100	–	0
Vancomycin	100	–	0
Erythromycin	90.3	3.2	6.5
Ceftriaxone	90.3	–	9.7
Ciprofloxacin	90.3	–	9.7
Clindamycin	87.1	9.7	3.2
Norfloxacin	87.1	–	12.9
Cotrimoxazole	71	–	29
Tetracycline	45.2	9.7	45.2

## Discussion

GBS is an important cause of infection in pregnant women and their newborns; however, there have been limited studies available in Ethiopia. Approximately one in five (19 %) pregnant women were colonized with GBS in the present study. In 11 (45.8 %) pregnant women GBS was isolated solely from rectal swabs. These rates of GBS would have been missed if only vaginal swabs were obtained, suggesting the importance of obtaining combined vaginal and anorectal swabs. This is consistent with the findings of other investigators who recommended combined vaginal and rectal swabs to maximize culture yield and increase detection rates [2, 19–21].

The rates of GBS colonization vary widely throughout the world. In Ethiopia, the study by Muhammed et al. (20.6 %) [11] showed GBS colonization rate that was higher than that reported by Schmidt et al. (9 %) [15]. The overall vaginal and anorectal colonization rate of 19 % in our pregnant population is high compared with the study conducted about 27 years ago among postpartum women in Gondar, Ethiopia, which was 9 % [15]. The differences in colonization rate might be due to geographic, as well as differences in the study participant (pregnant versus postpartum).

The prevalence of GBS colonization among pregnant women population has been documented in several countries, including Africa, Americas, Europe and Southeast Asia with the estimated mean prevalence rates ranging from 11.1 to 22.4 %. The lower prevalence of GBS colonization among pregnant women in Southeast Asia (11.1 %) compared to the present study may be related to regional differences in race and geographic area [10, 11, 22, 23]. The effect of race and geographical area on the prevalence of GBS colonization may be related to the differences in nutrition, socioeconomic status, sexually transmitted diseases, host immunity or sexual behavior [24, 25].

Knowledge about risk factors contributing for GBS colonization in pregnant women is relevant to minimize the morbidity, mortality associated with maternal and neonatal GBS infections [11]. In this study, no significant differences on the GBS colonization rate were detected when the socio-demographic and obstetric variables were considered. Unlike to our result, other studies showed that maternal age, education, gravidity and occupation have been observed as risk factors for GBS colonization among pregnant women [25–28]. However, there have been marked inconsistencies in studies that report the relationship between these factors and GBS colonization. For example, some studies showed that the prevalence of GBS increased with age [29], while others found it more with a younger age group [30, 31]. The findings from this study might be due to the small number of study subjects underpowered to find the possible risk factors, and these indicate the need for future large- scale study.

The universal prenatal screening and the risk-based approach are commonly used strategies proposed by the CDC in order to decrease the incidence of early-onset GBS neonatal infection [13, 14]. Before deciding which of these two strategies is to follow, a prevalence of GBS in the population has to be investigated (i.e., does the proportion of colonized pregnant women) with the identification of maternal risk factors.

In this study, all GBS isolates were uniformly sensitive to penicillin, ampicillin and vancomycin, which is consistent with previous reports recommending these antibiotics as the agent of choice for GBS prophylaxis [2, 11, 18, 29]. The proportion of isolates with in vitro resistance to clindamycin and erythromycin, which are considered first-line prophylaxis for those with an allergy to penicillin, has progressively increased since 1996 [14]. The prevalence of resistance among GBS isolates ranged from 7 to 40 % for erythromycin and from 3 to 26.4 % for clindamycin and might be associated with certain serotypes [19, 32, 33]. A relatively high level of resistance to tetracycline and cotrimoxazole in this study might be attributed to the wide and indiscriminate use of antimicrobial drugs. Since in Ethiopia, members from the public easily move into pharmacy shops to buy antibiotics without prescription (personal communication with drug vendors). This use of antibiotics is indiscriminate and might account for the high resistance rates observed.

## Conclusions and recommendations

This study reveals that one-fifth of pregnant women harbored GBS, which may pose a risk for the newborns. Therefore, further large-scale studies and data on the prevalence of GBS neonatal disease, preventative measures, and outcome of infected infants are imperative in this community to allow the preventive strategy. The study also indicates uniform susceptibility of GBS isolates from pregnant women to penicillin G, ampicillin and vancomycin. Even though no standard-of–care practices for GBS prevention in this setting, tetracycline and cotrimoxazole should not be used for GBS prevention. Future studies should be conducted to reveal serotype distributions of GBS in this community since monitoring of regional variations in serotype distribution is required to develop and implement effective vaccine for prevention of GBS disease.

## Abbreviations

GBS: group B *Streptococcus*
CDC: Centers for Disease Control and Prevention
N: number
SD: standard deviation
